# Challenging the osseous component of sphenoorbital meningiomas

**DOI:** 10.1007/s00701-019-04015-y

**Published:** 2019-08-01

**Authors:** Svenja Maschke, Mauricio Martínez-Moreno, Alexander Micko, Matthias Millesi, Georgi Minchev, Ammar Mallouhi, Engelbert Knosp, Stefan Wolfsberger

**Affiliations:** 1 Department of Neurosurgery, Medical University of Vienna, Waehringer Guertel 18-20, 1080 Vienna, Austria; 2 Department of Biomedical Imaging and Image-guided Therapy, Medical University of Vienna, Waehringer Guertel 18-20, 1080 Vienna, Austria

**Keywords:** Bone infiltration, Image guidance, Meningioma, Skull base, Sphenoorbital meningioma

## Abstract

**Background:**

Intraosseous growth is a unique feature of sphenoorbital meningiomas (SOM). Its close relation to neurovascular structures limits complete surgical resection and possibly contributes to the high recurrence rate.

**Objective:**

To evaluate the growth behavior of intraosseous remnants and develop a protocol for precise intraoperative visualization of intraosseous SOM.

**Methods:**

We included 31 patients operated for SOM from 2004 to 2017. The growth velocity of the intraosseous tumor component was volumetrically calculated in 20 cases. To improve accuracy of image guidance, we implemented a specialized bone surface-based registration algorithm. For intraoperative bone visualization, we included CT in multimodality continuous image guidance in 23 patients. The extent of resection (EOR) was compared with a standard MR-only navigation group (*n* = 8).

**Results:**

In 11/20 cases (55%), a progressive regrowth of the intraosseous SOM remnant was noted during a mean follow-up of 52 months (range 20–132 months). We observed a mean increase of 6.2 cm^3^ (range 0.2–23.7 cm^3^) per patient and side during the follow-up period. Bone surface-based registration was significantly more accurate than skin surface-based registration (mean 0.7 ± 0.4 mm and 1.9 ± 0.7 mm, *p* < 0.001). The EOR of the intraosseous component was significantly higher using CT + MRI navigation compared with controls (96% vs. 81%, *p* = 0.044).

**Conclusion:**

Quantitative assessment of the biological behavior of intraosseous remnants revealed a continuous slow growth rate independent of the soft tumor component of more than half of SOM. According to our data, application of a multimodal image guidance provided high accuracy and significantly increased the resection rate of the intraosseous component of SOM.

## Introduction

Apart from their dural component, sphenoorbital meningiomas (SOM) exhibit a unique intraosseous growth pattern within and adjacent to the sphenoid bone closely related to skull base neurovascular structures limiting surgical resection.

With up to 9% of all adult intracranial meningiomas [1, 2], SOM are not infrequent, and the vast majority is graded as WHO °I (84–100%) [3–5].

Tumor extension into the orbit results in the most common initial complaints of proptosis (86–93%) [5–7] and visual impairment (65–78%) [5, 6]; extension into the middle cranial fossa, cavernous sinus, and infratemporal fossa may cause later compressive symptoms.

The main goals of neurosurgical SOM treatment are improvement/prevention of visual impairment and reversal of exophthalmos by decompression of optochiasmatic and orbital structures from affected bone and soft tumor component. Albeit the intradural SOM being mostly amenable to extensive surgical removal, the dural involvement of orbital apex, superior orbital fissure (SOF), and cavernous sinus remains challenging. Concerning the intraosseous component of SOM, extensions medial to the cranial nerve ostia of the middle cranial fossa render a Simpson °I resection virtually impossible from a standard pterional approach. These growth characteristics contribute to the high recurrence rates of SOM after primary surgery, amounting to up to 60% after 5 years [8–11].

In the case of pure sphenoid wing meningiomas, recurrence rates were reported higher if bone infiltration was present (> 30% vs. 11.6%, respectively) [12]. In line with Simpson’s observations, a complete resection of the involved bone has been advocated to prolong progression-free survival in these meningiomas [2, 5, 13–15]. Regarded as a subtype of sphenoid wing meningiomas, this may also apply for SOMs.

Remnants of infiltrated bone are common after SOM surgery; however, the growth potential of the intraosseous SOM remnant remains unclear as it has not been systematically analyzed to date.

Microsurgical resection of osseous tumor extensions is impeded by the difficulty to visually distinguish osseous tumor from surrounding healthy bone intraoperatively. However, the extent of altered bone can easily be recognized in bone-windowed CT images.

The aims of the present study were (1) to evaluate the potential of regrowth of intraosseous remnants of SOM after subtotal resection, (2) to develop a protocol for precise intraoperative visualization of the intraosseous SOM component in relation to adjacent neurovascular structures, and (3) to assess whether the extent of resection (EOR) can be safely increased by the proposed method.

## Patients and methods

The study cohort comprises 31 patients with primary surgery for SOM between 2004 and 2017 in a tertiary care institution, i.e., 3.1% of all 1041 surgically treated intracranial meningiomas during this period.

Only cases with an attempted GTR were included. Planned partial resections, meningiomas located mainly beyond the confines of the sphenoorbital region, optic nerve sheath meningiomas, and meningiomatoses were excluded.

This study was approved by the ethics committee (EC no: 1144/2019).

### Patient and tumor characteristics

Clinical presentation: the presenting symptoms were extracted from the clinical information system, and patients were invited for follow-up interviews. Proptosis was assessed on preoperative CT images.

Histopathologic evaluation: the intraosseous tumor component was evaluated for meningioma infiltration. Meningioma specimens were examined for their histopathological type and WHO grade [16–18].

### Intraosseous growth assessment

CT scans are required to assess the extension of the osseous SOM component. All patients received CT scans within 48 h postoperatively and within the follow-up clinical examination of this study (additionally to the routine MRI follow-up examinations). As radiologic follow-up is usually performed by MRI that does not provide sufficient information on the evolution of intraosseous remnants, patients with a follow-up of >1 year were invited for CT scans.

An identical CT scanner and protocol was used for all scans (Siemens Somatom Sensation 64®, 0° gantry tilt, 1-mm spiral distance, 120 kV, 380 mA). Image manipulations and navigation guidance were all performed with a StealthStation S7 System (Medtronic, CO, USA). To determine the growth behavior of the intraosseous tumor component, the bone infiltration of the anterior and middle cranial fossa was first segmented semiautomatically. Then, the tumor volume was compared and the growth velocity calculated between early postoperative and follow-up CT scans.

### Definition of anatomical limits for safe resection

**Fig. 1:**
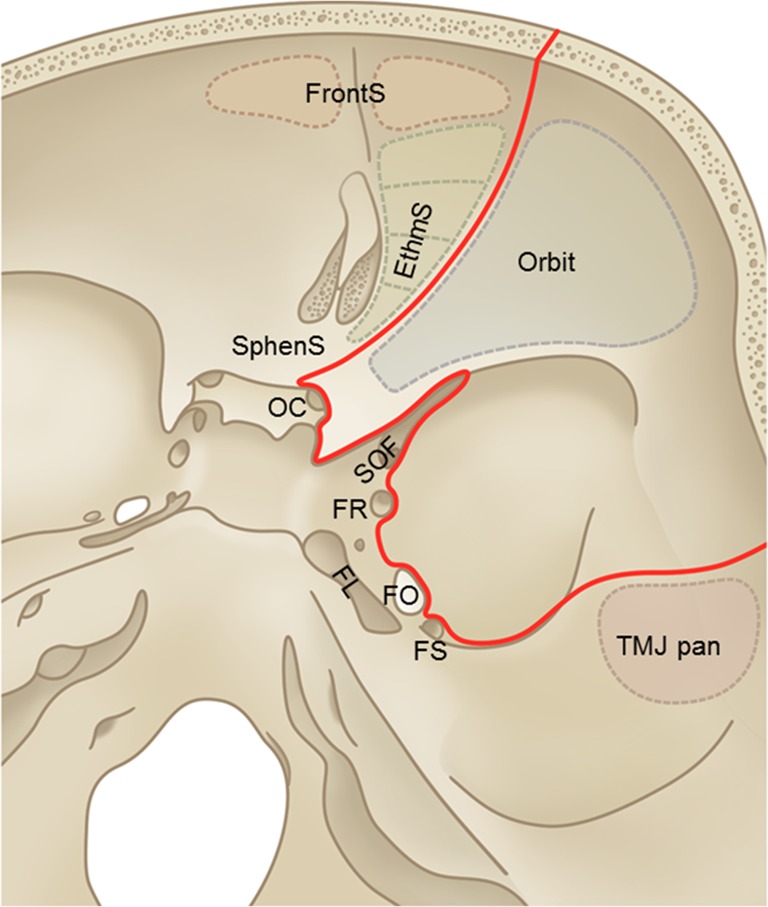
Neurovascular limit for safe resection of the intraosseous component of SOM. To safely accomplish removal of the largest part of the intraosseous tumor component, we propose an anatomical limit based on critical neurovascular structures. From a standard pterional approach, this limit was defined by the frontal, ethmoid, and sphenoid sinuses (FrontS, EthmS, SphenS), the medial wall of the optic canal (OC), superior orbital fissure (SOF), foramen rotundum (FR), foramen ovale (FO), foramen lacerum (FL), and temporomandibular joint (TMJ) pan. FS = foramen spinosum

To prevent neurovascular injury and sinunasal repair during a pterional approach, we propose anatomical limits to safely accomplish maximum SOM removal (Fig. 1):

for the intraosseous SOM component, this resection limit was defined medially by ethmoid and sphenoid sinuses, medial wall of the optic canal, SOF, foramina rotundum, ovale and lacerum, and the temporomandibular joint pan.

The soft tumor resection was limited by the contents of the SOF, orbital apex structures (annulus of Zinn), and cavernous sinus structures.

### Improvement of image guidance

#### Registration accuracy

**Fig. 2:**
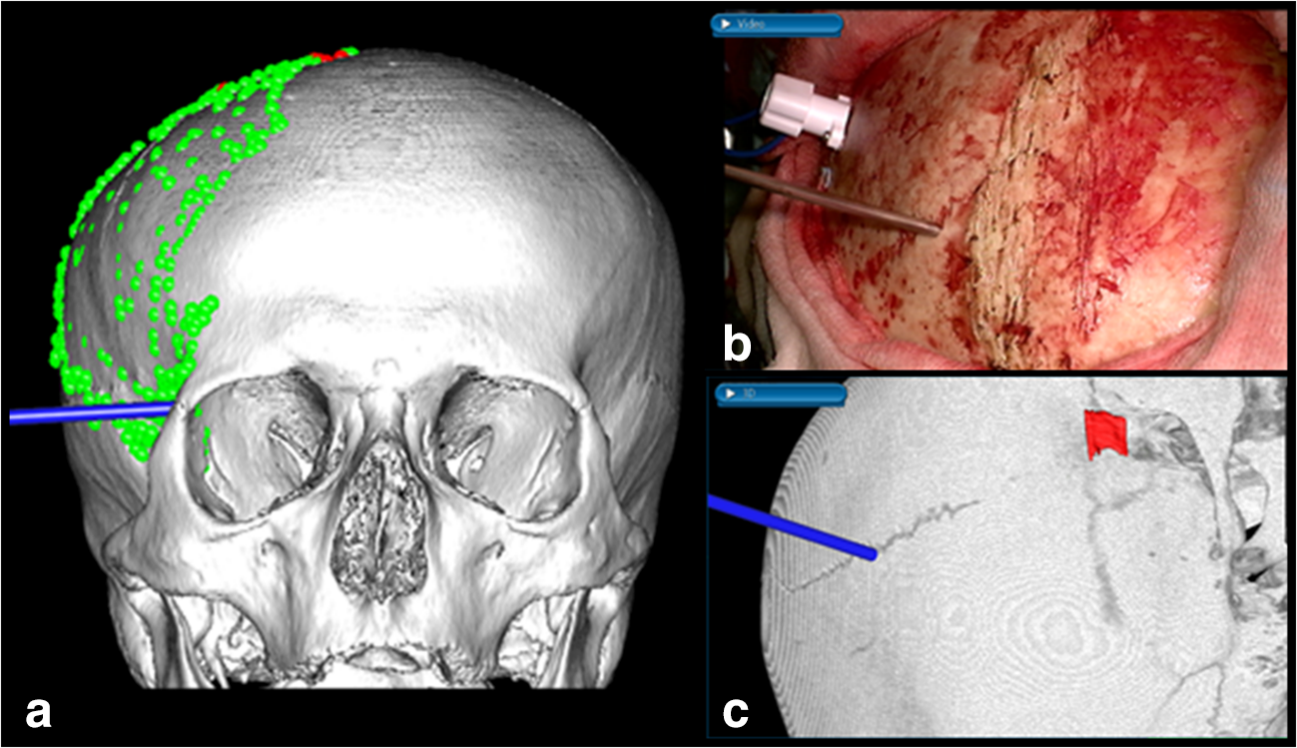
Bone surface-based registration: 3D-model used for registration (**a**). Concurrent intraoperative view of the exposed bone (**b**) with attached bone-attached patient reference tracker and landmark check of the coronal suture after bone surface-based registration (**c**)

A specialized bone surface-based registration algorithm was implemented to improve accuracy of image guidance for surgery of the osseous SOM component: preoperatively, a 3D skull surface model was created by threshold segmentation from CT data. After elevation of the skin flap, this model was registered to the exposed fronto-temporo-zygomatic bone surface, comparable with skin surface-based registration. Registration accuracy was checked on anatomical landmarks before proceeding with surgery (Fig. 2).

Prior to translation into the clinical setting, we compared feasibility and accuracy of this novel bone surface registration in a cadaver experiment with standard skin surface and bone screw registration.

#### Image guidance protocol

##### Bone-windowed CT scan

To improve visualization of the osseous SOM component, we merged a bone-windowed CT scan onto the routine contrast-enhanced T1-weighted MR (CE-T1MR).

Preoperatively, we semiautomatically segmented the osseous SOM component on the planning software. To facilitate identification of the infiltrated bone during surgery, the contours of the intraosseous SOM component were displayed in multiplanar views intraoperatively.

For higher accuracy, this CT scan (see imaging parameters above) was selected as registration reference [19].

##### Real-time tracking

To improve safety of removal of the osseous SOM component in the vicinity of neurovascular structures, we included real-time instrument tracking that provided continuous update of the resection progress utilizing instruments such as a navigated drill or tip-tracked suction device as described previously [19].

#### Group assignment

All 31 patients were operated by five experienced skull base surgeons. The intent of surgery was always the maximum removal of both soft and osseous SOM components. The impact of the addition of bone-windowed CT scan to MR image guidance (group CT + MRI, *n* = 23) on the extent of resection was compared with MRI-only navigation (group MRI, *n* = 8). The choice of the image guidance protocol was not based on tumor volume or extension but only on individual preference.

#### Extent of resection analysis

**Fig. 3:**
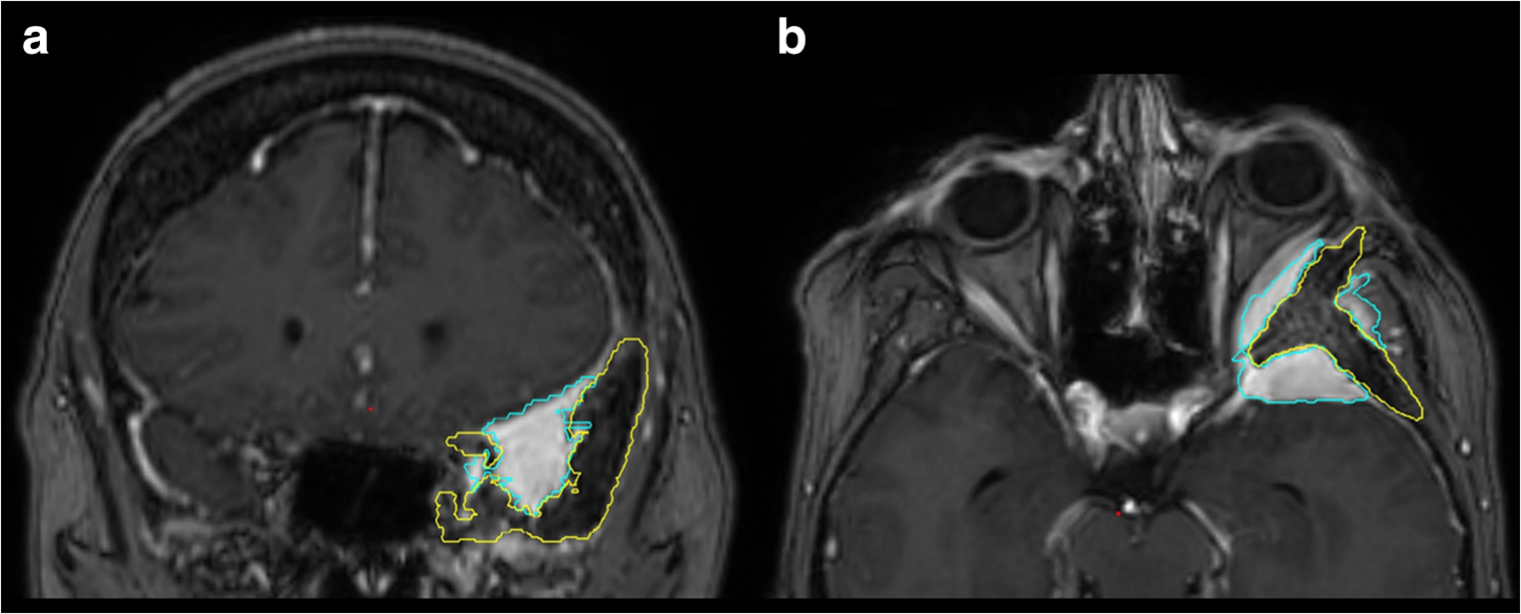
Case 49f, left-sided SOM: intraoperative multimodality image guidance concurrently displaying (1) osseous tumor extension (CT-based segmentation, *yellow*) and (2) soft tumor extension (MR T1 CE-based, *blue*). The background image is a MR T1 CE fat suppression MRI. **a** Soft tumor extension into the orbit via the SOF with surrounding osseous SOM involvement of the anterior clinoid process, the middle fossa floor extending up to the lateral wall of the sphenoid sinus, and the temporal bone. **b** Soft tumor extensions at the temporopolar dura, the SOF, the lateral orbit, and the temporal muscle. Osseous tumor extension in the greater wing of the sphenoid bone

Areas of osseous tumor involvement were defined pre- and postoperatively on CT-based volumetric studies. Tumor borders were segmented semiautomatically, and the tumor volume (cm^3^) was calculated. The soft tissue component of the SOM was identified separately in preoperative contrast-enhanced T1-weighted MRI fat suppression sequences (CE-T1MRI) in the same manner for all patients and postoperatively in eight patients who provided an MRI at follow-up (Fig. 3).

**Fig. 4:**
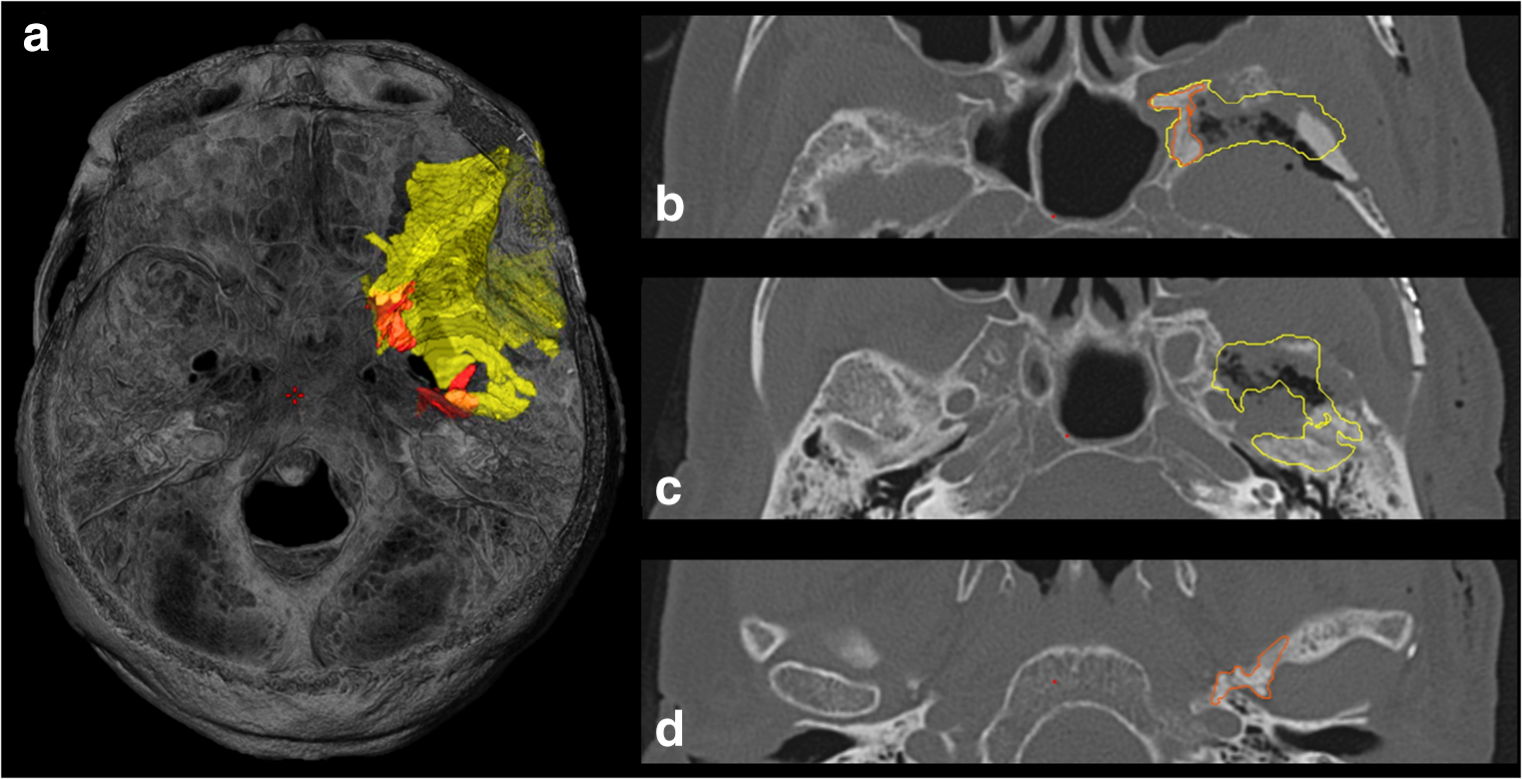
Operative result of case 54f by merging preoperative (*yellow*) and postoperative (*orange*) CT scans. **a** The osseous tumor remnants (*orange*) are mainly confined medial to the proposed line. **b** Removal of the lateral parts of the greater sphenoid wing. Resection was limited by involvement of the foramen rotundum and lateral sphenoid sinus wall. **c** Removal of the middle cranial fossa infiltrate lateral to the oval foramen. **d** Osseous remnant covering the TMJ pan medially and anteriorly

Residual tumor volumes of the intraosseous component (deemed resectable according to the line in Fig. 1) and of the soft tumor component were compared with preoperative tumor volumes in order to determine the EOR (Fig. 4).

### Statistical analysis

Variables are described as mean or median with range as appropriate. Mann-Whitney *U* test for differences in not normally distributed independent parameters between study and control group was applied. Asymptotic significances were chosen as two-tailed *p* values.

*p* values < 0.05 were considered significant. Analyses were carried out using SPSS software version 22.0 (IBM Corporation, USA).

## Results

### Clinical presentation and tumor characteristics (Table 1)

Patients presented average 14 months after onset of initial complaints (range 0–84 months).

**Table 1:** Patient and tumor characteristics

Patient characteristics	*n*	(%)
No. of patients	31	
Age (median, range)	51(38–82)	
Sex		
Female	27	(87.7%)
Male	4	(12.9%)
M:F ratio	1:6.75	
Side		
Right	15	(48.8%)
Left	13	(41.6%)
Bilateral	3	(9.6%)
Histopathological type		
Meningothelial °I	22	(71.0%)
Secretory °I	5	(16.1%)
Transitional °I	1	(3.2%)
Clear-cell °II	2	(6.4%)
Anaplastic °III	1	(3.2%)
Presenting symptoms		
Loss of visual acuity	14	(45.2%)
Temporal or lid swelling	8	(25.6%)
Lacrimation	6	(19.2%)
Headaches	5	(16%)
Conjunctival injection	5	(16%)
Double vision	4	(12.8%)
Ptosis	2	(6.4%)
Photopsia	1	(3.2%)

The most common presenting symptom in 27/31 (87%) patients was unilateral proptosis of mean 5.1 mm (range 1.4–8.5 mm) over the unaffected side (in the 28 cases of one-sided SOM) or 5.8 mm (range 4.4–7.8 mm) over the less affected side (in the three cases of bilateral SOM).

Decline of visual acuity was reported by 14 (45%) cases.

Surgical removal: all SOM resections were performed by standard microsurgical technique including high-speed diamond drill under neuronavigational guidance by senior surgeons.

For dural reconstruction, we used periosteum and/or synthetic dura substitute, for periorbital reconstruction fibrin-coated collagen fleece. Osseous defects were reconstructed with polymethylmethacrylate bone cement. Cosmetic results were satisfactory except for one case of postoperative enophthalmos.

Histopathological findings: all but three SOMs were classified as WHO °I—two were a clear-cell WHO °II and the other one an anaplastic WHO °III meningioma.

Meningioma infiltration was confirmed in the intraosseous SOM component in all patients (31/31).

Adjuvant treatments: the three cases of WHO °II and °III received postoperative radiation therapy. Six patients with WHO °I and progressive disease after reoperations received radiation therapy (*n* = 4) or radiosurgery (*n* = 2).

### Growth behavior of the intraosseous SOM component (Fig. 5)

For assessment of growth behavior of the intraosseous tumor component, our series comprised 25 patients with a minimum follow-up of 1 year. Of those, a follow-up CT scan was performed in 20 patients in the scope of this study. Of the remaining 5 patients, 3 were deceased (one of pancreatic cancer and two of unknown causes, including the patient with the WHO °III meningioma) and 2 were unavailable.

**Fig. 5:**
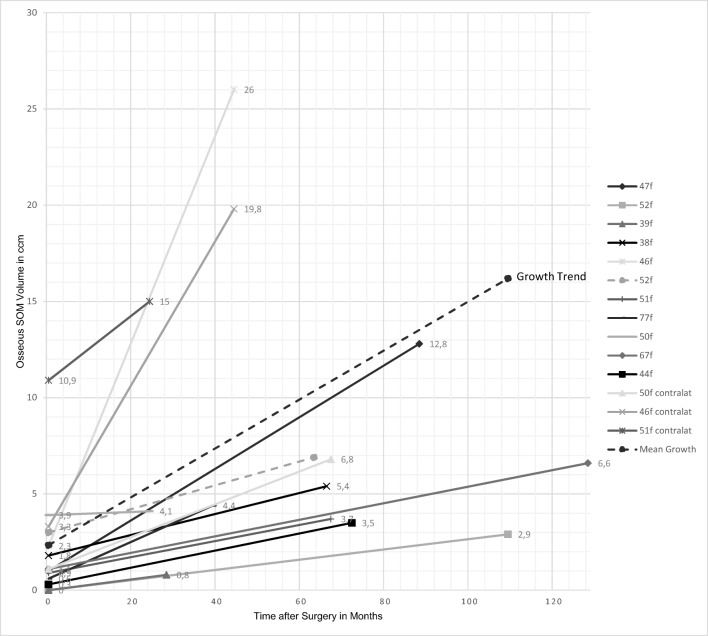
Growth rate of the intraosseous tumor component of SOM: in 11/20 cases (55%) with postoperative remnants and in all contralateral sides of the 3 bilateral tumors, a progressive regrowth was noted. Follow-up mean 52 months (range 20–132 months). Growth velocity mean 1.48 cm^3^ tumor increase per year (range 0.1–6.4 cm^3^/year), indicated by the interrupted line

During a follow-up of mean 52 months (range 20–132 months), progression of the intraosseous remnant was noted in 11/20 cases (55%) and an increase in volume of 6.2 cm^3^ (range 0.2–23.7cm^3^) per patient and side; all but one atypical meningioma were graded as WHO °I. Hence, growth trend of intraosseous SOM was mean 1.48 cm^3^/year (range 0.1–6.4 cm^3^/year). All three bilateral cases showed progression of tumor remnants as well as of the untreated side (Fig. 5).

In 9/20 cases (45%), no regrowth of intraosseous remnants was found during the mean follow-up of 56 months (range 20–174 months). Of these patients except for one WHO °II meningioma, all patients had WHO °I meningioma.

Of note, the mean follow-up did not differ significantly between regrowth and non-regrowth cases.

### Results of the image guidance protocol

#### Feasibility

Bone registration: in the lab setting, bone surface registration was significantly more accurate than skin surface registration (mean 0.7 ± 0.4 mm and 1.9 ± 0.7 mm, respectively, *p* < 0.001) approaching the submillimetric accuracy of skull screw registration (mean 0.3 ± 0.1 mm).

Clinically, the bone surface registration was feasible in all cases providing high accuracy and special usefulness during osseous tumor drilling. In two cases of limited exposure of the zygomatic arch, registration was successful at second repetition.

#### Extent of resection analysis (Table 2, Fig. 6)

Of all 31 patients, 23 (74%) were operated with the image guidance protocol; the remaining 8 (26%) operated with MR-only navigation served as controls. There were no statistically significant differences in preoperative tumor volumes of both the intraosseous (*p* = 0.206) and the soft tumor component (*p* = 0.414) between both groups.

**Table 2:** Extent of resection analysis

Tumor volumes (ccm)	Group CT + MRI	Group MIR	*p* value	*r* (Dohen)
Soft				
Preoperative	12.57 ± 29.1	7.81 ± 4.08	0.414	0.14
Postoperative	0.58 ± 1.18	2.05 ± 2.05	0.222	0.27
Resected	5.08 ± 4.41	6.50 ± 0.98	0.667	0.10
Intraosseous				
Preoperative	16.14 ± 8.80	19.41 ± 9.91	0.206	0.22
Postoperative	0.67 ± 1.04	3.57 ± 3.84	0.029	0.39
Resected	15.46 ± 8.68	15.83 ± 8.23	0.542	0.10
Total				
Preoperative	28.64 ± 29.22	31.15 ± 10.83	0.18	0.24
Postoperative	1.77 ± 1.44	6.00 ± 5.09	0.222	0.26
Resected	24.7 ± 15.58	31.05 ± 6.15	0.500	0.10
EOR (%)				
Soft tumor	96.11%	78.95%	0.286	0.25
Intraosseous tumor	95.80%	81.09%		0.36
Total tumor	88.46%	85.21%	0.500	0.15

**Fig. 6:**
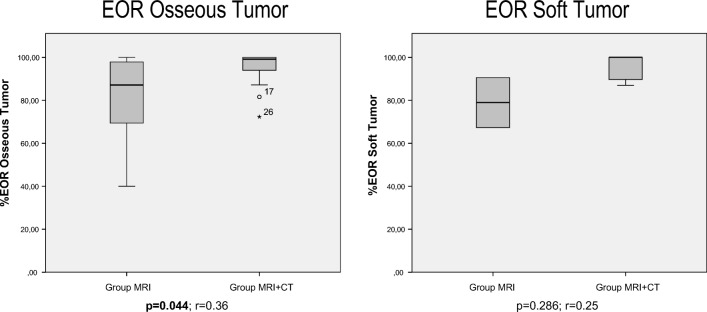
Comparison of EOR between CT + MRI and the MRI-only group. EOR was significantly higher for the osseous tumor component (*p* = 0.044; *r* = 0.36), but not for the soft tumor component (*p* = 0.286; *r* = 0.25) after application of the multimodality navigation protocol.

For the intraosseous tumor component, EOR was significantly higher using the navigation protocol compared with MR-only navigation (96% vs. 81% respectively, *p* = 0.044), but not for the soft tumor component.

We did not encounter increased operation times (mean duration 5.49 vs. 5.86 h for the multimodal image guidance vs. the MR-only navigation; *p* = 0.597), indicating a similar setup time.

#### Perioperative complications

There was one case of new postoperative unilateral amaurosis due to optic nerve compression by hemostyptic material irreversible despite emergency revision. We observed two transient and one permanent oculomotor and one transient trochlear nerve dysfunctions. Transient hypesthesia was reported for V1 in two, V2 in four, and V3 in one of the cases and permanent hypesthesia for V1 and V2 in two cases each. Permanent xerophthalmia by lacrimal gland/nerve dysfunction was reported by five patients.

We did not observe a significant increase in complication rate in the CT + MR group despite a more aggressive bone removal.

## Discussion

The growth potential of intraosseous remnants after incomplete SOM resection has not been systematically analyzed to date. As our data indicates a slow annual growth rate, maximum safe reduction of intraosseous SOM is warranted.

We propose an anatomical limit to which SOM can be safely resected. Further, we tested a novel image guidance protocol that was found to significantly increase the resection rate of intraosseous SOM.

### Osseous growth pattern

In the case of skull base meningioma with *sphenoid wing* origin, large intracranial tumors with some hyperostotic changes are regarded a distinct feature (> 90%) [20] and suggest osseous tumor invasion by infiltration of the Haversian system as first suggested by Echlin in 1934 [21, 22]. It still remains elusive to which extent other factors such as neoplastic enzymatic activity, osteoblastic stimulation by humoral factors, or vascular disturbances are involved [23–28]. The higher rate of recurrence after resection without the complete hyperostotic bone (Simpson ≥II) supports such osseous meningioma infiltration [6, 14, 29, 30].

In the case of *sphenoorbital* meningiomas, the extensive sphenoid osseous changes are the distinct feature [31]. In the case of absence of the dural component, other hyperostotic conditions such as primary intraosseous meningioma, fibrous dysplasia, Paget’s disease, and osteoma should be considered [30, 32].

The general hypothesis of SOM evolution is a primary dural origin with secondary osseous invasion [3]. There is a typical intradural temporopolar and periorbital growth of various globular sizes with possible *en plaque* extensions to the orbital roof and cavernous sinus. From the underlying intraosseous component, SOM may extend via sphenoid bone surfaces into temporal muscle and muscles of the infratemporal fossa or paranasal sinus mucosa as soft meningioma tissue. From these observations, a continuous growth pattern from dura via bone into extracranial soft tissues can be considered, but a primary sphenoid bone origin has not been ruled out.

Although there is no relation between the size of the tumor and the degree of hyperostosis [8, 31], the latter theory is supported by the observation that the intraosseous component can be disproportionately greater compared with the soft tumor component. This was reflected in our series with soft tumor volumes of mean 6.6 cm^3^ and osseous tumor volumes of mean 15 cm^3^.

Of note, the magnitude of the osseous invasion does not correlate with a more aggressive biological behavior [8].

Bilateral SOM are uncommon, yet three of our patients were found to have intraosseous and en plaque tumor components on both sides. Luetjens et al. proposed a staged resection with the more severely affected side treated first [33]. In our cases, only symptomatic sides and sides that increase in size were resected.

Our study shows that osseous remnants exhibit slow growth over the course of decades, and long-term follow-up is necessary for detection of clinical deterioration. One of our patients (not included due to lack of CT imaging studies in 1970s) with a 40+ year history of SOM developed a progressive exophthalmus and visual disturbances due to intraosseous tumor enlargement in the greater sphenoid wing with incipient narrowing of the orbital apex and was operated for recurrent SOM lately.

**Fig. 7:**
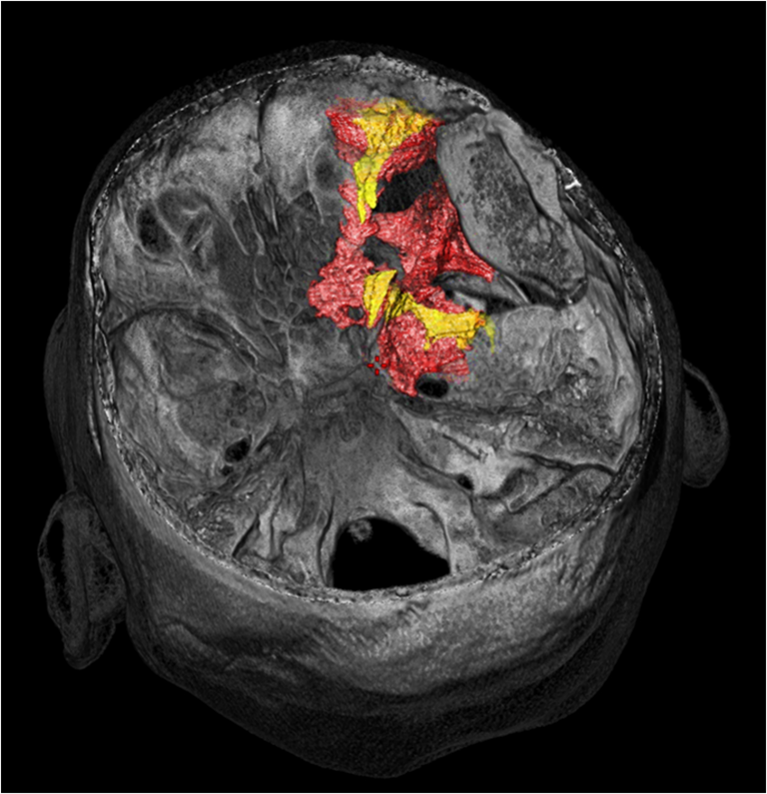
Example for regrowth of osseous SOM remnant. Case 47f, meningothelial SOM (WHO I): right-sided SOM presenting with exophthalmus and visual disturbance. At initial presentation, the intraosseous meningioma component was confined to lateral of the proposed resection line. A complete resection of the soft component and a subtotal resection of the osseous component with restitution of exophthalmus and vision was achieved (yellow = remaining osseous tumor, 1.1 ccm).

Therefore, maximum resection of the intraosseous SOM is warranted during primary surgery to prevent later inoperable infiltration of the skull base. This was highlighted by one case of our study that showed subsequent involvement of the frontal, ethmoidal, sphenoidal, and maxillary sinuses originating from a small postoperative remnant lateral to the proposed limit (Fig. 7).

In the cases of incomplete resection with osseous remnants, we suggest radiographic surveillance to early identify progression and potential soft tumor recurrence from these locations. The treatment armamentarium including reoperation, radiotherapy, and radiosurgery has to be individually tailored.

### Intraoperative image guidance

Intraoperatively, the osseous component of SOM is difficult to visually distinguish from healthy bone and does not respect osseous sutures. As it can be readily identified on bone-windowed CT, the inclusion of CT studies in image guidance to identify the intraosseous components has been proposed previously [34].

We advanced this concept by preoperatively segmenting intraosseous tumor areas on bone-windowed CT and intraoperatively displaying bony tumor extensions as colorized contours on the navigation screen.

Further, we applied tip-tracking of either drill or suction device as previously described [19], which allowed a seamless integration of navigation into the surgical workflow. Continuous instrument tracking was found useful in providing continuous information on the distance to neurovascular structures and osseous tumor borders. With the image guidance protocol, we were able to improve the EOR of the intraosseous component by 14%.

The duration of the resection was comparable between the study group and controls (5.4 h vs. 5.8 h; *p* = 0.59), while more tumor volume was resected with the image guidance protocol.

### Bone surface-based registration

The proximity of the intraosseous SOM component to neurovascular structures requires high navigational accuracy. In this study, we propose a bone surface-based registration as a highly accurate alternative to invasive bone screw-based [35, 36] registration or less accurate skin surface-based registration that has not been applied for surgery of SOM before.

For lateral otorhinolaryngologic skull base surgery with retroauricular bone exposure, surface matching of the temporal bone and mastoid has been proposed by Zhou et al. [37].

After ex vivo tests, we could show that this registration method is feasible for SOM resections and can be easily incorporated into the surgical workflow. Registration of the exposed skull before craniotomy averts loss of neuronavigational accuracy from positioning of the patient, draping and attachment of retractors [36]. SOM resections are especially suited for bone surface-based registration due to exposure of three dimensionally traceable structures mainly of the zygomatic bone.

### Anticipation of critical skull base structures

Visualization of modified bone may tempt the surgeon to extensive drilling, risking higher morbidity. We have therefore defined a resection margin at the skull base with medial limits at the cranial nerve foramina and ICA. Using continuous instrument navigation, the surgeon can anticipate these critical structures and duly limit tumor removal to avoid neurovascular injury.

Limited resection was also reported by other authors mainly inside the confines of the orbit around the SOF and the cavernous sinus [5, 38–41]. Adjuvant (stereotactic) radiation treatment/surgery has been applied in subtotally resected skull base meningiomas to control residual tumor and prolong progression-free survival [42, 43]. In the cases of extensive skull base involvement, however, our proposed protocol has the potential to further increase resection rates.

### Vascular Complications

Skull base surgery is fraught with injury to neurovascular structures. The risk of ICA injury can be minimized by anticipating the distance to the lacerum segment during drilling.

The ICA can be readily identified on CE-T1MRI, and CTA/MRA studies were not routinely included in the navigation protocol, but we recommend additional color coding of angiographic studies in selected cases with extensive bone infiltration of the middle cranial fossa floor to improve visualization of vascular structures and to avoid injury. The addition of angiographic images further aids in locating the middle meningeal artery for early tumor devascularization. In our series, no intraoperative ICA injuries were noted.

### Limitations of the study

#### Study design

Due to the rare incidence of SOM, the study design was retrospective. Due to the slow growing nature of the intraosseous SOM component, a more extensive follow-up duration of decades would be required for analysis of recurrence rates [11, 44].

#### Assessment of tumor growth

Intraosseous SOM growth was calculated from only two time points, subtracting early postoperative CT scan remnant volume from a recent CT scan acquired within the scope of this study. Since no assumptions can be made about individual growth curves from two time points alone, it is only a crude estimate to display the growth trend and not representative for volumetric extrapolation [45].

## Conclusion

Incomplete resection of the intraosseous component of sphenoorbital meningiomas is not infrequent. This first quantitative assessment of the biological behavior of intraosseous remnants revealed a continuous slow growth in more than half of the cases independent of the soft tumor component. Therefore, maximum safe reduction of the osseous tumor component is warranted but impeded by the proximity to neurovascular structures.

According to our data, addition of bone-windowed CT scan to image guidance was found to significantly increase the resection rate of the intraosseous component of sphenoorbital meningiomas.

## Abbreviations

CT: computed tomography
EM: electromagnetic
EOR: extent of resection
ICA: internal carotid artery
MR: magnetic resonance
SOM: sphenoorbital meningioma
SOF: superior orbital fissure
TMJ: temporomandibular joint
WHO: World Health Organization
